# Comparative Effects of Fructose and Glucose on Lipogenic Gene Expression and Intermediary Metabolism in HepG2 Liver Cells

**DOI:** 10.1371/journal.pone.0026583

**Published:** 2011-11-11

**Authors:** Kristin M. Hirahatake, John K. Meissen, Oliver Fiehn, Sean H. Adams

**Affiliations:** 1 Departments of Nutrition, University of California Davis, Davis, California, United States of America; 2 Molecular and Cellular Biology, University of California Davis, California, United States of America; 3 Genome Center, University of California Davis, Davis, California, United States of America; 4 Obesity and Metabolism Research Unit, USDA-Agricultural Research Service Western Human Nutrition Research Center, Davis, California, United States of America; Omaha Veterans Affairs Medical Center, United States of America

## Abstract

Consumption of large amounts of fructose or sucrose increases lipogenesis and circulating triglycerides in humans. Although the underlying molecular mechanisms responsible for this effect are not completely understood, it is possible that as reported for rodents, high fructose exposure increases expression of the lipogenic enzymes fatty acid synthase (FAS) and acetyl-CoA carboxylase (ACC-1) in human liver. Since activation of the hexosamine biosynthesis pathway (HBP) is associated with increases in the expression of FAS and ACC-1, it raises the possibility that HBP-related metabolites would contribute to any increase in hepatic expression of these enzymes following fructose exposure. Thus, we compared lipogenic gene expression in human-derived HepG2 cells after incubation in culture medium containing glucose alone or glucose plus 5 mM fructose, using the HBP precursor 10 mM glucosamine (GlcN) as a positive control. Cellular metabolite profiling was conducted to analyze differences between glucose and fructose metabolism. Despite evidence for the active uptake and metabolism of fructose by HepG2 cells, expression of FAS or ACC-1 did not increase in these cells compared with those incubated with glucose alone. Levels of UDP-N-acetylglucosamine (UDP-GlcNAc), the end-product of the HBP, did not differ significantly between the glucose and fructose conditions. Exposure to 10 mM GlcN for 10 minutes to 24 hours resulted in 8-fold elevated levels of intracellular UDP-GlcNAc (P<0.001), as well as a 74–126% increase in FAS (P<0.05) and 49–95% increase in ACC-1 (P<0.01) expression above controls. It is concluded that in HepG2 liver cells cultured under standard conditions, sustained exposure to fructose does not result in an activation of the HBP or increased lipogenic gene expression. Should this scenario manifest in human liver *in vivo*, it would suggest that high fructose consumption promotes triglyceride synthesis primarily through its action to provide lipid precursor carbon and not by activating lipogenic gene expression.

## Introduction

Understanding the etiology and consequences of obesity in the context of the current nutritional environment could lead to prevention and intervention methods that will greatly improve metabolic health outcomes. The deleterious consequences of disorders prevalent in obesity including dyslipidemia, consisting of hypercholesterolemia (high LDL), low HDL, and hypertriglyceridemia, have made obesity a major national public health concern. The increasing trend of overweight, obesity and related metabolic disease is thought to be the result of dietary changes (e.g., increased calorie intakes coupled to nutrient-poor, calorie-rich foods) that have not been countered by adequate physical activity. While not proof of causation, the historic obesity prevalence trend parallels the increase in dietary sugar and high fructose corn syrup (HFCS) consumption by the U.S. population, leading some to propose that fructose intake has played a role in driving positive energy balance and overweight [1]. Furthermore, compared with glucose, high fructose intake is associated with an enhanced rate of *de novo* lipogenesis (DNL) and increased circulating lipids in humans [2], [3], with the caveat that most experiments have been conducted at very high levels of dietary sugar intake. In human subjects fed high fructose diets over 10 weeks, hepatic DNL was increased significantly compared to glucose [4]. Furthermore, the prevalence and severity of non-alcoholic steatohepatitis appears to be higher in persons consuming high fructose diets [5], [6], [7]. The liver is the primary site of DNL [8] and is responsible for the utilization of most of the dietary fructose arriving via the portal vein, on the order of >70% [9], [10], [11]. However, the exact molecular mechanisms that underlie increased hepatic lipid production following consumption of high fructose diets are not clear.

Current evidence indicates that the lipogenic nature of fructose is largely due to the availability of hepatic triose-phosphates and pyruvate following fructose consumption that serve as precursors for fatty acid synthesis, as reviewed by Havel [12]. Phosphorylation of fructose to fructose-1-phosphate (F-1-P) by the enzyme fructokinase (KHK) promotes direct flow of fructose carbon into the glycolytic pathway, bypassing a key regulatory enzyme of glycolysis, phosphofructokinase (PFK). For this reason, a higher proportion of the carbon from ingested fructose, as compared with glucose, is metabolized into triglycerides [12]. Another possible explanation for the increase in DNL seen in response to high fructose consumption is increased expression and activation of components of the lipogenic enzymatic pathway in the liver. It is well documented that feeding of high sucrose or high fructose diets (or drinking high levels of these sugars) to mice and rats increases the mRNA levels and enzymatic activities of the key lipogenic enzymes fatty acid synthase (FAS) and acetyl-CoA carboxylase (ACC-1, or ACCα) in the liver [13], [14], [15], [16], [17], [18]. Several investigators have found that fructose feeding in rodents results in increased FAS activity and hepatic triglyceride content when compared with isocaloric diets of glucose, sucrose, or starch [14], [19], [20]. High fructose feeding to rodents has also been shown to raise the hepatic mRNA levels of transcription factors, including sterol regulatory element binding protein-1 (SREBP-1) and carbohydrate response element binding protein (ChREBP), involved in the regulation of genes encoding glycolytic and lipogenic enzymes [18], [21], [22]. However, it is unknown whether such gene-level changes occur in human liver in response to increased consumption or tissue exposure to fructose, and the specific metabolic signals that trigger lipogenic gene expression in response to fructose or sucrose remain to be identified.

The metabolic and lipogenic enzyme expression outcomes observed with high fructose feeding in rodents are strikingly similar to those resulting from activation of the hexosamine biosynthesis pathway (HBP), a minor branch from glycolysis whose initial substrate is fructose-6-phosphate (F-6-P). For instance, it has been demonstrated that chronic flux through the HBP in transgenic mice overexpressing glutamine∶fructose-6-phosphate amidotransferase (GFAT, the rate-limiting enzyme of the HBP) leads to insulin resistance and hyperlipidemia [23], [24]. In isolated adipocytes from GFAT transgenic mice, glucosamine (GlcN) that directly enters the HBP at the level of GlcN-6-P up-regulated mRNA levels for FAS and ACC-1 in a dose-dependent manner and to a greater extent than glucose [25]. The overexpression of GFAT in human HepG2 liver cells resulted in 19-fold and 7-fold increases in FAS and SREBP-1 transcript levels, respectively, and these effects were abolished by treatment with a GFAT inhibitor [26]. The HBP has been characterized as a mediator of nutrient sensing and metabolic regulation, and the major end product of the HBP, UDP-*N*-acetylglucosamine (UDP-GlcNAc), can react with serine or threonine residues of nuclear and cytosolic proteins. Increasing the UDP-GlcNAc pool results in enhanced *O*-linked glycosylation of transcription factors, such as Sp-1, involved in the regulation of gene expression by nutrients [27], [28]. Thus, increased flux through the HBP, as expected following a glucose-rich meal or under hyperglycemic conditions, would in theory promote lipogenic gene expression to accommodate energy storage as lipid. It remains to be established whether activation of the HBP is a physiologic regulator of lipogenic genes in human liver.

In the current experiments, we investigated the hypothesis that high fructose exposure would increase hexosamine generation in human hepatocytes, and that this increase would be associated with increased lipogenic gene expression. The rationale for this hypothesis is as follows. In the liver, the first step of glucose metabolism involves its phosphorylation to glucose-6-phosphate (G-6-P) by hexokinases (HKs), after which the G-6-P is further metabolized to either G-1-P (precursor for glycogen synthesis) by phosphoglucomutase or to F-6-P by phosphoglucose isomerase. The F-6-P enters glycolysis, the pentose phosphate pathway, or the HBP as a hexosamine precursor via conversion to GlcN-6-P by GFAT. In theory, fructose could also trigger formation of F-6-P by hexokinases traditionally associated with glucose metabolism. For instance, it was shown that HK2 and HK4 are capable of phosphorylating fructose directly to F-6-P: Katzen et al. determined the K_m_ for glucose and fructose of rat HK2 to be 1.4×10^−4^ M and 3.0×10^−3^ M, respectively [29], and Malaisse et al. reported that 25% of hepatic fructose in rats is phosphorylated to F-6-P by HK4 [30]. Studies have also shown that glucose confers kinetic cooperativity to human liver and β-cell hexokinase toward fructose phosphorylation to F-6-P [31], [32]. Finally, it is possible that active fructose metabolism to triose-phosphates could increase the intracellular G-6-P pool, and hence promote F-6-P generation and HBP flux through attenuation of glucose catabolism and not as a direct F-6-P precursor. To test the hypothesis that fructose activates lipogenic enzyme expression associated with an increase in HBP metabolites, a human HepG2 liver cell model was used to determine detailed temporal patterns of FAS and ACC-1 expression coupled to intracellular metabolite profiling following fructose treatment compared with the effects measured in cells incubated in glucose alone.

## Materials and Methods

### Materials

MEM and MEM non-essential amino acids were purchased from Cellgro (Manassas, VA), fetal bovine serum (FBS, Lot # 60816) was purchased from Atlas Biologicals (Fort Collins, CO), and cell culture plates were purchased from BD Falcon (Franklin Lakes, NJ). A human total RNA tissue panel was obtained from Clontech (Mountain View, CA; Cat #636643). Antibodies were purchased from Cell Signaling Technology, Inc. (Danvers, MA).

### Cell Culture

HepG2 cells (a human hepatocarcinoma cell line; ATCC HB-8065) were cultured in MEM (Minimum Essential Medium Eagle) containing 10% (v∶v) FBS, 100 U/mL penicillin and 100 µg/mL (Invitrogen, Carlsbad, CA), 1× MEM non-essential amino acids, and 5.5 mM glucose. Cells were maintained at 37°C in a 5% CO_2_/air environment. Cells were grown to between four and six passages in 10 cm tissue culture dishes with 14 mL MEM, and transferred to MULTIWELL™ 6 well culture dishes for incubation in 3 mL of the treatment medium. Upon reaching 80% confluency, the cell culture medium was changed to 5.5 mM glucose MEM plus 5 mM fructose, or 5.5 mM glucose MEM plus 10 mM GlcN. The control plates received fresh 5.5 mM glucose MEM. These conditions allowed for exposure of liver cells to a physiological concentration of glucose, similar to what would be anticipated *in vivo* where the liver is never exposed to fructose alone.

### Gene Expression Analysis

mRNA was prepared from cells grown for 24, 48 or 72 hours in the respective treatments. This experiment was repeated three times with n = 2/treatment per experiment, to give a total of n = 6 per treatment group. Total RNA was extracted using a RiboPure® Kit (Ambion, Austin, TX) per manufacturer's protocol. RNA abundance and integrity were checked using a NanoDrop® ND-1000 Spectrophotometer (NanoDrop Technologies, Wilmington, DE) and an Agilent 2100 bioanalyzer (Agilent, Foster City, CA) per manufacturer's instructions. One microgram of total RNA was reverse transcribed into cDNA using the SuperScript® III reverse transcriptase (Invitrogen) followed by RNase –H treatment as per manufacturer's instructions. The quantitative real-time PCR assays utilized gene-specific TaqMan® primers and FAM-MGB labeled probes (Assays-on-Demand®, Applied Biosystems, Foster City, CA) and were analyzed in triplicate for each sample using an ABI 7900HT instrument. Reactions were carried out in a 384-well format containing the following in each well: cDNA corresponding to 20 ng of original total RNA, 1× specific primer probe mix and 1× Master Mix (ABI Taqman Gene Expression Master Mix); cDNA was air dried in each well prior to adding qPCR reagents to facilitate an 8 µL/well assay. Cycle conditions were 50° C for 2 minutes, 95°C for 10 minutes, then 40 cycles of 95°C for 15 s/60°C for 1 minute. Amplification cycle threshold number (Ct) of housekeeping mRNA (HPRT1) for each sample was determined using commercial HPRT1 primers and probes (ABI, Hs99999909_m1 ) to correct for template loading differences across all target genes (ΔCt = target gene Ct−reference gene Ct), and expression values were determined relative to treatment control transcript levels using a mathematical formula previously described [33]. Amplicon sizes were checked by high resolution 3% MetaPHOR® agarose gel (Cambrex Bio Science Inc., Rockland, ME). Primer/probe ABI identifiers for gene expression studies were FASN (Hs01005622_m1), ACACA (Hs01046047_m1), KHK (Hs00240827_m1), SREBP1 (Hs01088691_m1), ChREBP (Hs00263027_m1), GCK (Hs01564555_m1), HK2 (Hs00606086_m1), SLC2A2 (Hs01096904_m1), and SLC2A5 (Hs00161720).

### Western Blot Analysis

Following exposure to 5.5 mM glucose-containing MEM alone (controls) or 5.5 mM glucose MEM plus 5 mM fructose or 10 mM glucosamine for 48 hours, HepG2 cells (n = 6/treatment) were washed once with HBSS, lysed with M-PER (mammalian protein extraction reagent) with 1× HALT protease and phosphatase inhibitors (Thermo Fisher SC), and sonicated for 10 s. Lysates were centrifuged at 20,000 g at 4°C for 10 min. Protein concentrations were quantitated using the bicinchoninic protein assay (Pierce). For FAS and ACC-1, 30 µg of total protein was separated on a 3–8% Tris-Acetate gel using Tris-Acetate SDS running buffer (Invitrogen). The proteins were transferred to a polyvinylidene diflouride membrane and immunoblotted with rabbit anti-FAS or anti-ACC-1 antibody (1∶1000) in PBST for 1 hour at room temperature. Specific signal was detected with a horseradish peroxidase-conjugated secondary antibody (1∶5000 goat anti-rabbit HRP) using Immun-Star™ WesternC™ Kit (BioRad Laboratories, Hercules, CA). Blots were imaged using a ChemiDoc™ XRS+ Imaging System (BioRad). β-actin was used as a loading control.

### Fructose and Glucose Utilization Analyses

Changes in the fructose and glucose concentrations in aliquots from the culture media containing 5.5 mM glucose alone or with 5 mM fructose after HepG2 incubation for 24 hour periods were determined using modification of a commercial glucose/fructose analytical kit (Megazyme, catalog item K-FRUGL; Xygen Diagnostics Inc., Burgessville, Ontario, Canada) as previously described [10].

### Lactate Production

Lactate production was measured by quantifying the amount of lactate in the conditioned media using a modification of the assay methodology for the SYNCHRON® System (Beckman Coulter Inc., Brea, CA). The assay employs the conversion of lactate to pyruvate and hydrogen peroxide (H_2_O_2_) by lactate dehydrogenase, followed by peroxidase-facilitated reaction of H_2_O_2_ with dichlorobenzenesulfonic acid (DCBSA) and 4-aminoantipyrine to form a chromophore that is measured spectrophotometrically at 512 nm. The reaction conditions were: 10 µL sample added to 200 µL of assay reagent (100 mM Tris (pH 7.4), 700 U/L lactate oxidase, 1000 U/L peroxidase, 2 mM DCBSA, 1.16 mM 4-aminoantipyrine) and incubated in the dark for 10 minutes before analysis.

### Metabolite Profile Analysis

For pilot work to establish conditions for cellular metabolite analysis over time, HepG2 cells (5×10^5^ cells/well) were cultured in MULTIWELL™ 12 well culture dishes containing 2 mL/well of 5.5 mM glucose MEM, MEM+5 mM fructose, or MEM+10 mM GlcN. Media was replenished at 24 hr and at 48 hr, after which cell extractions were carried out after 10 min, 60 min, 6 hr, or 24 hr. For extraction, culture plates were immediately placed on ice and each well was rinsed twice with 1 mL ice-cold PBS and then 4 mL ice cold 3∶1 methanol/H_2_O solvent was added (assuming the ratio 1 mL solvent/2 mg tissue based on pilot studies measuring cell pellet weight/well). Cells, along with the extraction reagent, were scraped into 5 mL collection tubes kept on ice and the samples were stored at −80°C before analysis.

Cellular metabolite concentrations were determined using either GC-TOF (fructose, G-6-P, F-1-P, and F-6-P) or LC-MS (GlcN-6-P and UDP-GlcNAc). For the GC-TOF and LC-MS analyses, samples were thawed on ice and vortexed for 20 s, sonicated continuously for 5 min (VWR 50HT Ultrasonic bath), and separated into 500 µL aliquots. Each aliquot was centrifuged for 5 min @ 14,000 g, and the supernatant was collected and lyophilized to dryness. Samples were kept on ice whenever possible, removed only for sonication, centrifugation, and lyophilization steps. The GC-TOF protocol and procedure then followed a method previously described [34]. For the LC-MS analysis lyophilized material was re-dissolved in 100 µL initial LC gradient solvent and analyzed within 24 hr. Chromatography was performed on an Agilent 1200 Series HPLC system (Agilent Technologies, Santa Clara, CA). Samples were housed in an autosampler maintained at 4°C, and 5 µL of material was injected on a Luna 3 µm NH_2_ 2.0×150 mm column (Phenomenex, Torrence, CA). Column temperature was kept at 40°C. Mobile phase consisted of H_2_O with 10 mM ammonium acetate and 10 mM ammonium hydroxide (A) and 9∶1 acetonitrile/H_2_O with 10 mM ammonium acetate and 10 mM ammonium hydroxide (B). The gradient method was: 0–2 min at 50% B, 2–5 min of a linear gradient to 0% B, and 5–20 min at 0% B. The column was re-equilibrated for 15 min following each sample separation, and flow rate was constant at 0.4 mL/min. LC eluents were analyzed with an Agilent 6530 accurate-mass Q-TOF mass spectrometer equipped with an Agilent Jet Stream ESI source in negative ion mode. MS data was collected with a 0.25 sec scan rate in both profile and centroid modes, and mass calibration was maintained by constant infusion of reference ions at 112.9856 and 980.0164 m/z. MS/MS data generation was targeted for GlcN-6-P. The parent ion m/z was isolated between 7.5–12.5 min with a 1.3 m/z isolation width and a constant collision energy of 15 eV.

These experiments confirmed that, under the cell culture conditions used for gene expression work, detection of targeted metabolites could be readily achieved and changes in analyte concentrations could be observed over time (see Results and Figures S1, S2, and S3). Experiments to test for metabolite profiles in glucose vs. fructose-treated HepG2 cells mimicked the pilot work but also included a treatment with 10.5 mM glucose to match the total sugar concentration of the MEM+5 mM fructose treatment.

### Statistics

Data were analyzed using Prism software version 5.02 (GraphPad, San Diego, CA). Values in the text are means ± S.E.M. One-way ANOVA followed by Dunnett's multiple comparison test was used for multiple comparison studies within a treatment timepoint. Two-way ANOVA was used for metabolite analyses; comparing time, treatment, and time×treatment interactions. A P value of 0.05 was considered significant. Bartlett's test for variance homogeneity was used to determine that variance was equal within each experiment. Each experiment was repeated a minimum of 3 times, and the sample sizes per treatment are indicated in the text, figure legends and tables.

## Results

### Characterization of carbohydrate metabolism capacity in HepG2 cells

To establish that HepG2 cells are a valid model for studying fructose metabolism, we first confirmed the expression of relevant transporters and enzymes required for liver carbohydrate catabolism. The abundances of glucose transporter 2 (GLUT 2, primary liver glucose and fructose transporter), GLUT 5 (fructose transporter), HK2, HK4 (glucokinase, GCK), and fructokinase (KHK) mRNAs in cells grown in standard 5.5 mM glucose MEM were compared with that of a commercially-available human tissue panel (**Table 1**). Quantitative real-time PCR results showed expression of all key components in HepG2 cells except HK4. In human liver, HK4 was detectable, yet not before 30 PCR cycles. To ascertain if our treatments impacted the expression of these genes in HepG2 cells, further experiments were conducted to examine the mRNA levels of these targets following incubation in the three types media after 24, 48 and 72 hours (**Table 2**). Incubation of the cells in 5.5 mM glucose MEM plus 5 mM fructose did not significantly affect the level of gene expression of these components at any time point compared with control cells grown in 5.5 mM glucose MEM, with the exception of 24 hr when GLUT2 expression was lower in the fructose-treated group. In cells treated with MEM plus 10 mM GlcN, GLUT5 transcript levels were increased at 48 and 72 hr and HK2 expression was increased at the 24 hr timepoint.

**Table 1:** mRNA expression profile of transporters and enzymes important for simple carbohydrate catabolism in HepG2 cells and human tissues using real-time PCR.

			Gene		
	GLUT 2	GLUT 5	HK 2	GCK	KHK
Tissue		% of human liver mRNA abundance			
Liver	100	100	100	100	100
HepG2	67	132	14262	ND	175
Dorsal Root Ganglion	≤1	1759	201	≤1	10
Spleen	ND	387	272	≤1	≤1
Stomach	≤1	360	484	2	4
Bone Marrow	ND	6824	1287	ND	≤1
Heart	≤1	938	497	≤1	2
Kidney	29	14374	85	≤11	396
Lung	ND	209	984	ND	4
Placenta	ND	776	994	ND	≤1
Skeletal Muscle	ND	11259	2661	≤1	2
Spinal Cord	≤1	10960	1188	9	9
Testis	≤1	64189	10151	5	17
Uterus	ND	331	141	≤1	4
Colon	ND	2069	6975	≤1	14
Pancreas	≤1	22.1	28	14	2
Small Intestine	21	26820	110	≤1	97
Fetal Liver	33	191	182	ND	21
Fetal Brain	≤1	1467	478	4	17
Adrenal Gland	≤1	493	199	2	4
Brain	≤1	1494	311	9	16
Cerebellum	ND	75	1077	ND	5
Prostate	≤1	6702	807	≤1	2
Salivary Gland	ND	112	225	≤1	2
Thymus	≤1	661	477	2	≤1
Thyroid	≤1	197	837	20	2
Trachea	≤1	209	592	ND	2

GLUT 2 (SLC2A2), solute carrier family 2 member 2; GLUT 5 (SLC2A5), solute carrier family 2 member 5;

HK2, hexokinase 2; GCK, glucokinase (hexokinase 4); KHK, ketohexokinase (fructokinase).

ND = Not detected.

**Table 2:** mRNA expression of carbohydrate transporters and catabolic enzymes in HepG2 cells cultured in standard MEM (control, containing 5.5 mM glucose), MEM+5 mM fructose, or MEM+10 mM glucosamine (GlcN) for 24, 48, and 72 hours.

			Gene		
Time		GLUT 2	GLUT 5	HK 2	KHK
24 Hours				% of Control	
	Control	100.0±4.6	100.0±11.8	100.0±8.6	100.0±5.1
	5 mM Fructose	92.0±2.6	88.9±19.4	104.7±13.9	80.0±7.8
	10 mM GlcN	78.5±5.1*	159.7±25.8	157.7±20.6*	97.3±5.5
48 Hours					
	Control	100.0±5.2	100.0±10.5	100.0±6.9	100.0±1.0
	5 mM Fructose	103.9±10.3	103.1±12.9	70.0±12.8	84.9±7.3
	10 mM GlcN	100.6±16.3	171.8±29.3*	72.0±14.7	103.7±14.3
72 Hours					
	Control	100.0±3.6	100.0±1.9	100.0±1.7	100.0±0.6
	5 mM Fructose	121.5±7.2	123.2±15.3	89.9±21.9	103.1±9.6
	10 mM GlcN	91.72±13.8	192.2±9.3*	180.5±53.5	109.0±14.7

Values are means ± SEM, n = 5 or 6/group.

*P<0.05 vs. control.

GLUT 2 (SLC2A2), solute carrier family 2 member 2; GLUT 5 (SLC2A5), solute carrier family 2 member 5; HK2, hexokinase 2; KHK, ketohexokinase (fructokinase).

Note: GCK (HK4) was not detected in any samples.

The HepG2 cells actively metabolized fructose, as 24 hr conditioned media from cells exposed to 5.5 mM glucose MEM+5 mM fructose revealed substantial decreases in media fructose concentrations over time (**see Fig. S1**), which equates to significant net fructose uptake in the cells. Uptake of fructose from the media was 3.8±0.5, 6.8±1.3, and 7.0±0.9 µmol/24 hr at the 24, 48, and 72 hr treatment timepoints, respectively. Net uptake of glucose by cells grown in media containing fructose was similar to controls, with 10.8±1.1, 11.6±0.6, and 13.6±0.3 µmol glucose/24 hr at the 24, 48, and 72 hr treatment timepoints; for comparison, control cells grown in 5.5 mM glucose MEM utilized 9.5±0.7, 12.0±0.2, and 14.1 µmol glucose/24 hr at each of the respective timepoints, and these values were not significantly different compared to fructose-treated cells. Cells grown in control 5.5 mM glucose MEM produced 5.0±0.8, 4.7±0.4, and 6.4±0.9 µmol lactate/24 hr at 24, 48, and 72 hours of treatment, values that were not significantly different compared to cells grown in glucose plus fructose: 5.9±0.2, 5.2±0.8, and 7.1±1.3 µmol lactate/24 hr and 24, 48, and 72 hours, respectively.

### Effects of fructose on lipogenic gene expression

**Figure 1:**
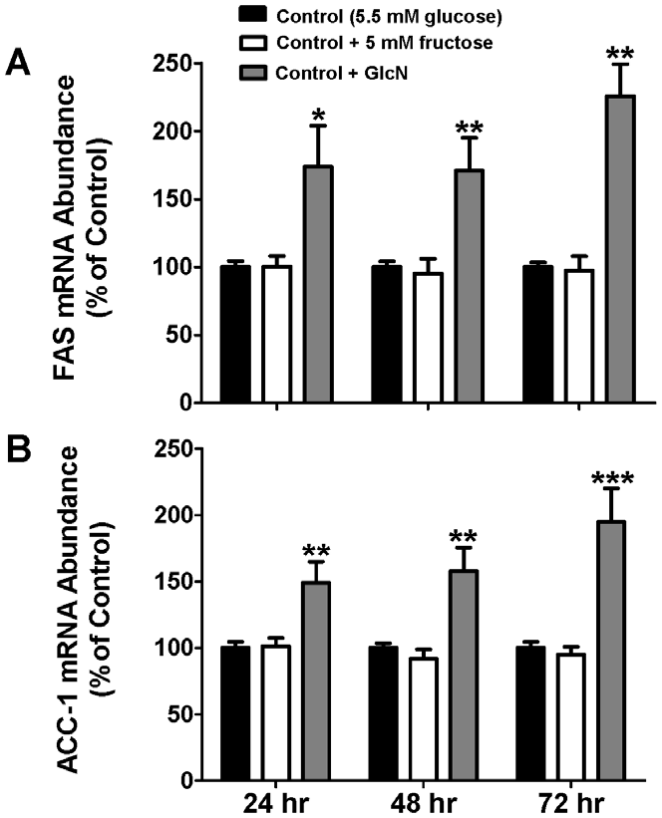
Induction of lipogenic gene expression by glucosamine (GlcN) treatment but not fructose treatment of HepG2 cells. Transcript abundances of (**A**) FAS (fatty acid synthase) and (**B**) ACC-1 (acetyl-CoA carboxylase) at 24, 48 and 72 h was determined in cells incubated in 5.5 mM glucose (control), glucose+5 mM fructose, or glucose+GlcN. mRNA abundance is expressed relative to the mean level of expression of the control samples within each time point. Means ± SEM are depicted. *P<0.05, **P<0.01, ***P<0.001 vs. controls; n = 5 or 6 for each group.

**Figure 2:**
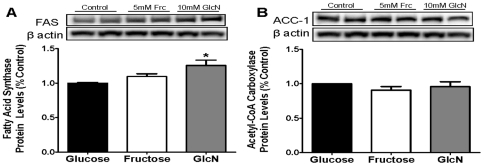
Elevation of FAS but not ACC-1 protein levels by GlcN treatment in HepG2 cells. Protein levels of key lipogenic enzymes (**A**) FAS and (**B**) ACC-1 in HepG2 cells incubated for 48 hours in media containing 5.5 mM glucose, glucose+5 mM fructose, or glucose+10 mM GlcN. Significant increase in FAS protein levels of HepG2 cells exposed to 10 mM GlcN relative to control was seen. There was no statistical difference between the protein levels of ACC-1 in any of the treatment groups. *P<0.05 vs. controls. Means ± SEM are depicted; n = 6 for each group.

Quantitative RT-PCR results demonstrated a significant increase in the abundance of key lipogenic enzyme mRNA levels in HepG2 cells cultured in 10 mM GlcN-enriched medium for 24, 48, and 72 hours compared with those grown in control or fructose-enriched medium (**Fig. 1A and 1B**). Transcript levels of FAS were increased up to 125.7±23.6% compared to the glucose-only control group and ACC-1 mRNA abundance increased up to 95.0±25.2% relative to controls. A follow-up study using earlier 1 and 6 hr endpoints (n = 4/group) did not reveal any change in FAS or ACC-1 gene expression among the conditions. Contrary to our hypothesis, the addition of 5 mM fructose to the culture medium produced no induction of FAS or ACC-1 mRNA above controls. The mRNA levels of the transcription factors SREBP-1 and ChREBP did not differ significantly across the three groups (data not shown). Additionally, we examined whether the protein levels of FAS and ACC-1 corresponded with the gene expression results seen after 48 hr of treatment. Cells treated with GlcN exhibited a significant increase in FAS protein, however there was no effect of fructose or glucose treatments (**Fig. 2A**). Differences in ACC-1 protein across treatments were not observed (**Fig. 2B**).

### GC/MS and LC/MS metabolite profiles of HepG2 cells

To determine if fructose consumption by HepG2 cells resulted in an increase in cellular HBP metabolites, metabolite-profiling studies were conducted in cells grown 5.5 mM glucose alone or 5.5 mM glucose plus 5 mM fructose. Cells cultured in the hexosamine precursor GlcN (5.5 mM glucose MEM+10 mM GlcN) were used as a positive control for HBP metabolites. Pilot studies (see Materials and Methods; n = 2/treatment) confirmed that under the cell culture conditions and extraction protocol employed, HBP metabolites including UDP-*N*-acetylglucosamine (UDP-GlcNAc) and GlcN-6-P as well as several carbohydrate metabolites of interest (e.g., fructose, F-6-P, F-1-P and G-6-P) could be readily detected. Preliminary data from this analysis showed high levels of UDP-GlcNAc, the major end product of the HBP, in the GlcN treated cells (see **Fig. S2**). Interestingly, GlcN-6-P levels were trace to non-detectable under all conditions, suggestive of rapid flux of precursor metabolites toward UDP-GlcNAc. As expected, high amounts of cellular fructose were detected in the cells exposed to fructose-enriched medium, whereas only trace amounts were seen in the other groups. For instance, in fructose treated cells, the level of fructose (average quantifier peak ion height) 10 min after fresh medium was 19,743 and increased to 26,406 after 1 hr. The fructose concentration decreased over the next two timepoints (**Fig. S3A**). Cellular F-6-P was also readily detectable at all timepoints and under all treatment conditions (**Fig. S3B**). F-1-P was not measured in this preliminary analysis. These findings established the time frame and conditions that would be used for the broader study, and provided initial preliminary evidence for a lack of effect of fructose on HepG2 HBP metabolites.

**Figure 3:**
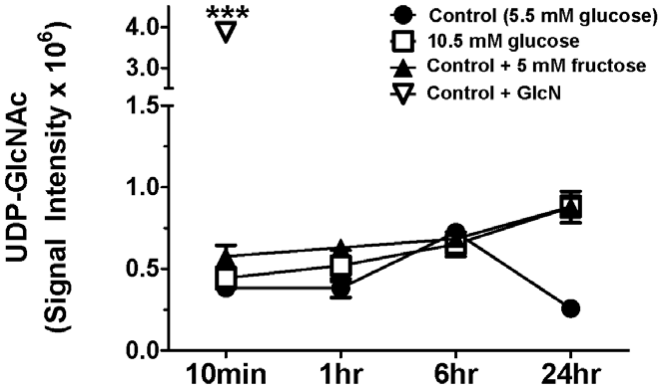
Cellular concentrations of UDP-GlcNAc in HepG2 cells following exposure to glucose and fructose or glucose alone. LC-QTOF results for cellular levels of UDP-GlcNAc in HepG2 cells at 10 min, 1 hr, 6 hr, or 24 hr time points following the addition of culture medium. The 10 mM GlcN condition, serving as a positive control, showed significant induction of the hexosamine biosynthesis pathway (HBP). Unit of intensity is quantifier ion peak height. Means ± SEM are depicted. ***P<0.001 vs. controls; n = 3–6/group.

Having established the conditions for the larger experiment, we chose to harvest cells after 10 min exposure to 10 mM GlcN to again validate that HepG2 cells demonstrate HBP activity and that these downstream metabolites could be detected. Consistent with the results of the pilot data, incubation of HepG2 cells in 10 mM GlnN-enriched medium resulted in the robust accumulation of cellular UDP-GlcNAc (**Fig. 3**). The abundance of UDP-GlcNAc in cells exposed to GlcN was 7 to 10 times greater than cells grown in the other three conditions. There was no significant difference in the amount of this metabolite in cells grown in 10.5 mM glucose and 5.5 mM glucose plus 5 mM fructose at any time point. At 24 h, there was significantly less UDP-GlcNAc detected in the 5.5 mM glucose condition, although this is expected due to the lower concentration of total sugar in the media over time as glucose was consumed.

**Figure 4:**
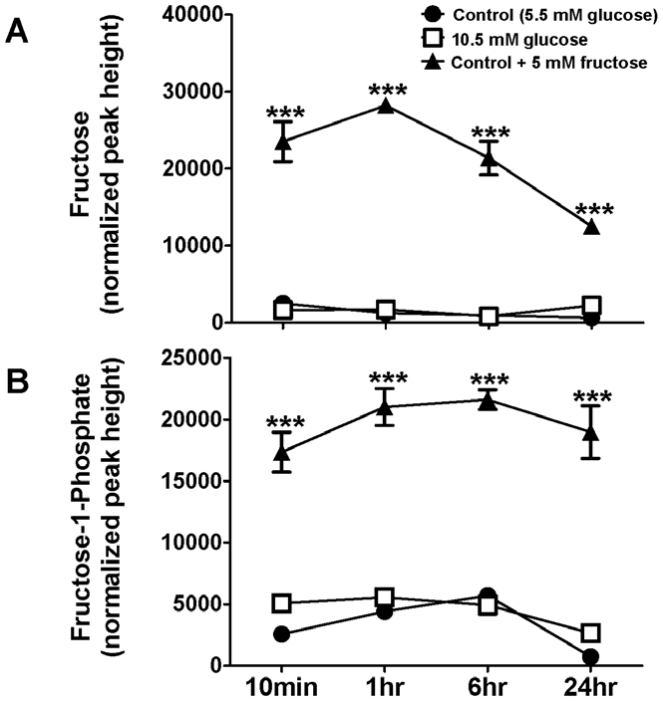
Concentrations of fructose metabolites in HepG2 cells incubated in the presence of glucose and fructose or glucose alone. (A) Cellular levels of fructose in HepG2 cells grown in 5.5 mM glucose (control), 10.5 mM glucose, or 5.5 mM glucose+5 mM fructose for 48 hours before new media was added for 10 min, 1 hr, 6 hr, or 24 hr. (B) Levels of fructose-1-phosphate (F-1-P) in HepG2 cells grown in the three conditions described above. Units of intensity are quantifier ion peak heights. Means ± SEM are depicted. ***P<0.001 vs. controls; n = 5 or 6/group.

This set of experiments provided further confirmation of the active uptake and metabolism of fructose from the media by HepG2 cells. Cellular fructose levels reached a peak 10 min to 1 hr following the addition of new fructose-enriched media (**Fig. 4A**). Evidence of fructose metabolism is clear from the elevated amounts of F-1-P found in the cells incubated in fructose-containing medium, compared with the glucose groups (**Fig. 4B**).

**Figure 5:**
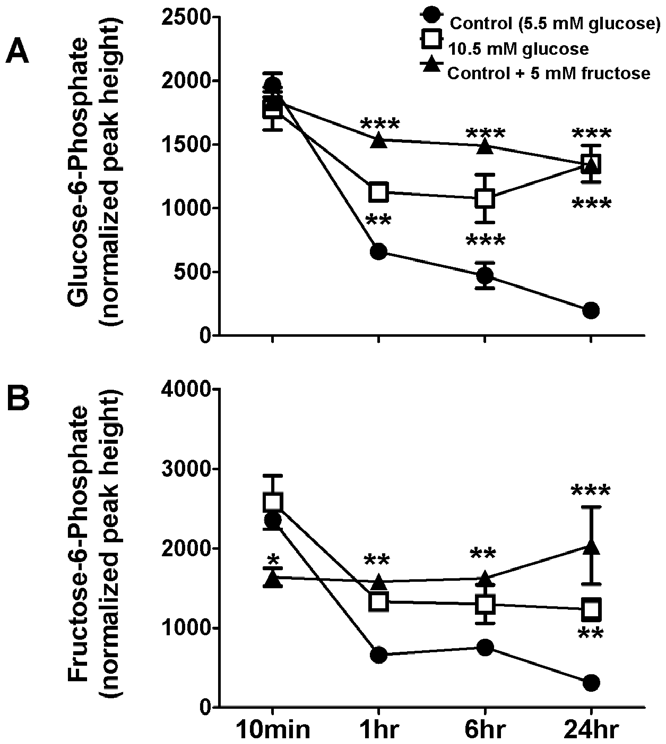
Changes in hexose-6-phosphate levels in HepG2 cells grown in glucose or fructose. (**A**) Abundance of G-6-P in HepG2 cells grown in 5.5 mM glucose (control), 10. 5 mM glucose, or glucose+5 mM fructose for 48 hours before new media was added for 10 min, 1 hr, 6 hr, or 24 hr. (**B**) Levels of fructose-6-phosphate (F-6-P) in HepG2 cells grown in the three conditions described above. Units of intensity are quantifier ion peak heights. Means ± SEM are depicted. *P<0.05, **P<0.01, ***P<0.001 vs. controls; n = 5 or 6/group.

Several interesting trends were also seen in primary metabolites of glucose metabolism. In HepG2 cells grown in both 5.5 mM and 10.5 mM glucose, the abundance of G-6-P was highest 10 min after fresh media was added and decreased progressively over time (**Fig. 5A**). Cellular levels of G-6-P in fructose-treated cells also follow a downward trend over the course of 24 hr. However, despite media glucose uptake equivalent to cells grown in standard 5.5 mM glucose MEM (see above), cellular G-6-P levels in the fructose group remained significantly elevated over those of cells grown in standard MEM throughout the time course. Similar findings were seen in the cellular concentrations of F-6-P, a metabolite classically thought to be produced primarily by the isomerization of G-6-P via phosphoglucose isomerase. HepG2 cells grown in either concentration of glucose alone exhibited a decrease in the cellular abundance of F-6-P over time (**Fig. 5B**), consistent with the diminishing precursor pool (G-6-P). This trend was more pronounced in the 5.5 mM glucose condition. In contrast, levels of F-6-P in cells grown in MEM plus 5.5 mM fructose remained constant over time.

## Discussion

The increase of lipogenesis and circulating triglycerides following high intake of fructose or sucrose in humans is well established [4], [35], [36], [37], but the underlying molecular mechanisms driving this effect are not completely understood. The canonical model of hepatic fructose metabolism is that fructose is readily metabolized in the liver to F-1-P via the action of KHK (fructokinase) and this leads to entry of lipogenic carbon into the glycolytic cycle below the key glycolysis regulatory point PFK [11], [38]. Our current metabolite profiling studies are consistent with this since the concentration of F-1-P was rapidly and significantly increased in HepG2 cells grown in a fructose-enriched media. Questions that have remained unanswered, however, are whether or not acute or chronic hepatic fructose exposure can predispose toward a lipogenic state through activation of lipogenic enzyme expression and if so, what are the metabolite signals involved? It is well-documented in animal models that the consumption of large amounts of fructose is associated with increases in liver expression of lipogenic FAS and ACC-1 enzymes [3], [15], [18], [19]. Should this take place in human liver upon chronic excessive dietary fructose intake, it would have important implications for lipid homeostasis since elevated DNL in the basal state and following sugar intake would be expected as a result of increased lipogenic enzyme capacity. Considering the logistical and ethical difficulties in obtaining liver biopsies from human subjects consuming high glucose or high fructose diets, the HepG2 cell model provides an important tool to understand how fructose and glucose differentially and temporally impact intermediary metabolism and gene-level events in human hepatocyte-derived cells.

It was anticipated that active fructose uptake and catabolism in HepG2 cells would yield increased expression of FAS and ACC-1 reminiscent of what is observed in rodent liver. However, despite demonstration that HepG2 cells express transporters and hexokinases capable of driving fructose utilization, and clear evidence from metabolite profiling studies that these cells actively take up and catabolize fructose, incubation of HepG2 cells with this sugar did not lead to induction of lipogenic gene or protein expression when compared with glucose alone. In contrast, Collison et al. reported increases of ACC-1 gene expression following treatment of HepG2 cells with 2.5% HFCS [3], but interpretation is confounded since the concentration of fructose was not reported but can be estimated to be exceptionally high and unphysiological at >30 mM. Following a fructose-enriched meal, systemic plasma fructose concentrations increase to ∼0.5 mM [37] and portal vein concentrations are certainly much higher considering that the liver consumes ∼50–80% of blood fructose per pass [10], [11]. Thus, the fructose concentration chosen for the current study (initially 5 mM and decreasing to ∼2.5 mM over time) ensured adequate sugar availability to the cells at levels expected to be at the upper end of postprandial portal venous concentrations. Based on our results, we conclude that active metabolism of fructose is not sufficient to induce the lipogenic machinery in HepG2 cells. Should this manifest in human liver, it suggests that increases in DNL *in vivo* following high intakes of dietary fructose, HFCS, or sucrose [4] may be attributed primarily to increased generation of fructose-derived lipogenic precursor carbon and not from activation of DNL enzyme expression. It is acknowledged, however, that interpretations based on a hepatoma cell line must be viewed with caution, and cell culture experiments cannot always mimic the specific hormonal and metabolite milieu of the liver *in vivo*. Studies designed to determine the effects of fructose-containing diets on human liver lipogenic gene and protein expression *in situ* are thus warranted, especially at varying dietary intake levels that span the range seen in the population.

We hypothesized that high fructose exposure would drive accumulation of intracellular F-6-P due to direct phosphorylation of fructose by hexokinases and/or through inhibition of normal glucose glycolytic catabolism due to feedback inhibition by fructose-derived downstream metabolites (e.g., citrate inhibition of PFK). Indeed, unlike cells treated with 5.5 mM glucose alone, cellular levels of F-6-P remained stable and were not reduced over time in cells incubated with 5.5 mM glucose plus fructose. Since F-6-P is the first committed metabolite in the hexosamine biosynthetic pathway (HBP), it was anticipated that fructose could activate the HBP in HepG2 cells. It has previously been reported that activation of the HBP through overexpression of the HBP enzyme GFAT in HepG2 cells [26] or through provision of the HBP precursor GlcN to liver and adipose cells [25], [39] can induce lipogenic gene expression. Consistent with the latter report, we observed that GlcN treatment of HepG2 cells significantly increased mRNA expression of FAS and ACC-1, concurrent with higher cellular concentrations of UDP-GlcNAc. However, despite a relative maintenance of G-6-P and F-6-P concentrations in HepG2 cells treated with fructose vs. those treated with glucose alone, there was no evidence for induction of the HBP pathway by fructose treatment. It is important to note that the nature of the pathway responsible for converting F-6-P to hexosamines in human liver is not well characterized, as most studies of hepatic hexosamine biology have been done in genetically manipulated cells or animal models. It may be that flux through the HBP from catabolism of sugars is too low to impact lipogenic gene expression in human hepatocytes, in contrast to GlcN that directly enters the HBP pathway and results in a dramatic increase of hexosamine formation. GFAT is expressed in human liver [40] and HepG2 cells [26], but its actual enzyme activity in these cells is not known. The fact that fructose failed to increase HBP metabolite pools above that of glucose-treated HepG2 cells might explain the lack of change of lipogenic gene expression in response to this sugar.

Another interesting observation concerning cellular F-6-P dynamics was that despite a more stable and maintained F-6-P concentration in HepG2 cells provided fructose compared to those given glucose alone, immediately after provision of fresh sugar-containing culture media, F-6-P levels were significantly higher in cells provided glucose compared to those given fructose plus glucose (see 10 min time point, **Fig. 5**). The underlying explanation for this remains elusive. Perhaps in cells provided glucose only, exposure to glucose after a relative depletion phase rapidly expands the glucose-derived G-6-P pool and thus drives isomerization to F-6-P through phosphoglucose isomerase. Possibly, in cells consuming fructose alongside glucose, very acutely there could be a lower initial rate of extracellular glucose flux to intracellular F-6-P compared to cells consuming glucose alone, and this may be coupled to a relatively limited production of F-6-P from direct phosphorylation of fructose.

Metabolite profiling revealed evidence that fructose metabolism attenuates glycolytic flux of glucose in HepG2 cells. In cells treated with 5.5 mM glucose alone, there was a drop in cellular G-6-P levels over time, likely explained by the ∼70% reduction of media glucose substrate over 24 h in culture. We suspect that this reflects a decrease in the media concentration of glucose below the K_m_ of GLUT 2, the primary glucose transporter in the liver. In contrast, cells grown in 10.5 mM glucose sustained a higher concentration of cellular G-6-P over time, probably due to higher media glucose levels throughout the incubation period. In cells grown in 5.5 mM glucose plus fructose, the maintenance of cellular G-6-P concentration resembled that of cells incubated in 10.5 mM glucose. Since glucose uptake was the same comparing cells grown in 5.5 mM glucose with those grown in 5.5 mM glucose plus fructose, this may reflect a blockade in glycolysis and subsequent maintenance of the cellular G-6-P pool in cells provided fructose. These observations are consistent with the concept that triose-phosphates generated from fructose metabolism increase the quantity of fructose carbon that enters the glycolytic pathway distal to PFK. These intermediates are ultimately metabolized to citrate, which can limit glucose metabolism through feedback inhibition of PFK [12].

In conclusion, the current findings indicate that like human liver *in vivo*, HepG2 cells actively utilize fructose, and temporal metabolite profiling highlights that fructose exposure rapidly increases cellular fructose, F-1-P, and G-6-P pools and maintains the cellular F-6-P level. Despite active metabolism of fructose, there was no evidence for activation of hexosamine biosynthesis or induction of lipogenic gene expression. The data from HepG2 cells are consistent with the hypothesis that increased DNL and blood VLDL triglyceride levels typically observed following acute and chronic high fructose intake *in vivo* primarily result from robust hepatic generation of lipogenic precursor metabolites, and not from specific induction of the lipogenic enzyme machinery in human liver. If true, the latter may result from a low capacity for hexosamine production from F-6-P in human hepatocytes. Our findings indicate a need for further studies to elucidate the relationship between fructose consumption, hepatic DNL, and hexosamine production in human liver *in situ*.

## Supporting Information

Figure S1 **Decrease in fructose concentration in HepG2 fructose-enriched culture medium following 24 hr incubation periods.** The change in fructose concentration in 5.5 mM MEM supplemented with 5 mM fructose after HepG2 incubation for 24, 48, and 72 hr. Media was replenished every 24 hr. Means ± SEM are depicted; n = 6 or 12/group.
(TIF)

Figure S2 **Elevated cellular concentrations of UDP-GlcNAc in HepG2 cells exposed to GlcN, but not glucose or fructose.** Preliminary GC-TOF results quantifying UDP-GlcNAc levels in cells grown in 5.5 mM glucose, glucose+5 mM fructose, or glucose+10 mM GlcN for 1 hr following 48 hr incubation in treatment media. Unit of intensity is quantifier ion peak height. Means ± SEM are depicted; n = 2/group.
(TIF)

Figure S3 **High levels of intracellular fructose and temporal changes in fructose-6-phosphate (F-6-P) in HepG2 cells grown in fructose-enriched medium compared with glucose and GlcN.** Pilot study GC-TOF data showing (**A**) elevated cellular fructose levels and (**B**) relatively higher maintenance of F-6-P concentration in cells grown in 5.5 mM glucose+5 mM fructose for 10 min to 24 hr following 48 hr incubation in treatment medium. Units of intensity are quantifier ion peak heights. Means ± SEM are depicted; n = 2/treatment per time point.
(TIF)
