# Supplementary figures and images for: Comparative Effects of Fructose and Glucose on Lipogenic Gene Expression and Intermediary Metabolism in HepG2 Liver Cells
Source: PLoS One. 2011 Nov 11;6(11):e26583. doi: 10.1371/journal.pone.0026583 (PMC3214012; doi:10.1371/journal.pone.0026583)

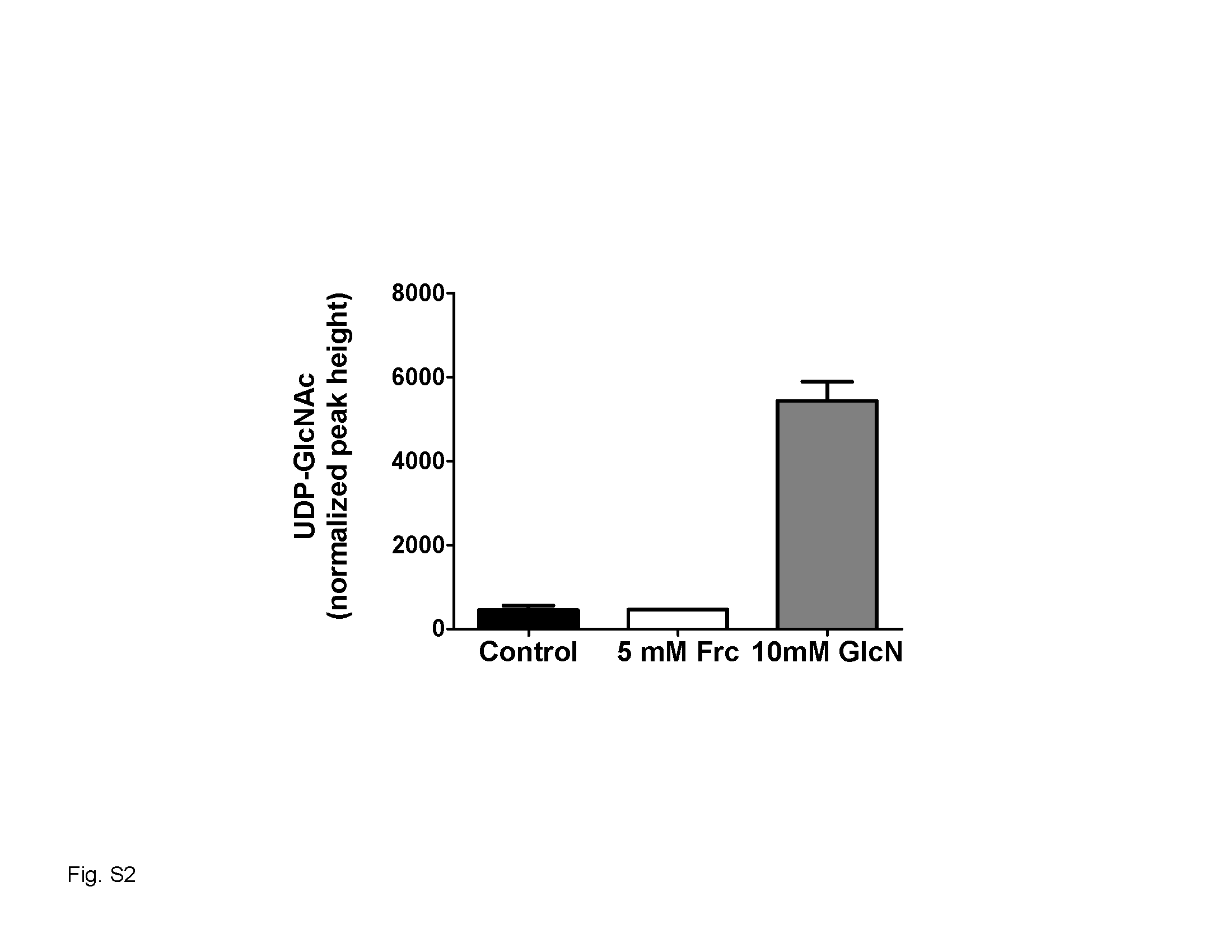

Supplement: Figure S2 — Elevated cellular concentrations of UDP-GlcNAc in HepG2 cells exposed to GlcN, but not glucose or fructose. Preliminary GC-TOF results quantifying UDP-GlcNAc levels in cells grown in 5.5 mM glucose, glucose+5 mM fructose, or glucose+10 mM GlcN for 1 hr following 48 hr incubation in treatment media. Unit of intensity is quantifier ion peak height. Means ± SEM are depicted; n = 2/group. (TIF) [file pone.0026583.s002.tif]
